# First Experience with Fluorescence in Pediatric Laparoscopy

**DOI:** 10.1055/s-0039-1692191

**Published:** 2019-07-05

**Authors:** Beatriz Fernández-Bautista, David Peláez Mata, Alberto Parente, Ramón Pérez-Caballero, Juan Carlos De Agustín

**Affiliations:** 1 Department of Pediatric Surgery, Hospital General Universitario Gregorio Maranon, Madrid, Spain; 2 Department of Cardiovascular Surgery, Hospital General Universitario Gregorio Maranon, Madrid, Madrid, Spain; 3 Department of Pediatric Surgery, Gregorio Marañon University Hospital, Madrid, Spain

**Keywords:** indocyanine green, laparoscopy, fluorescence, thoracoscopy, minimally invasive surgery

## Abstract

**Background** The use of intraoperative fluorescence images with indocyanine green (ICG) has recently been described as an aid in decision-making during surgical procedures in adults.

We present our first experiences with different laparoscopic procedures performed in children using ICG fluorescence images.

**Material and Method** We have used ICG fluorescence imaging technique in varicocele ligation, two nephrectomies, cholecystectomy, and one case of aortocoronary fistula closure. All procedures were performed through a minimally invasive approach. A high definition camera equipped with a visible infrared light source and gray-scale vision technology was used.

After injection of ICG before or during the laparoscopic procedure, precise identification of vascular anatomy and bile duct architecture were easily identified. Fluorescence helped to assess blood flow from the spermatic vessels, define the variability of renal vascularization, and determine the precise location of the aortocoronary fistula. Biliary excretion of the ICG allowed the definition of the biliary tract.

**Conclusion** Fluorescein-assisted images allowed a clear definition of the anatomy and safe surgical maneuvers during surgical procedures. The ICG imaging system seems to be simple and safe. Larger and more specific studies are needed to confirm its applicability, expand its indications, and address its advantages and disadvantages.

## Introduction

Since the 1980s, minimally invasive surgery has provided technological advances in different areas of surgery.

The current use of new techniques has allowed the recent introduction of indocyanine green (ICG), which has facilitated the approach and the prevention of intraoperative complications in adults. It provides greater clarity and depth image visualization and reduces surgical time. 1 Regarding cholecystectomy, it helps to better identify the bile duct anatomy, and in case of urological or oncological surgery, it allows to define the vascular anatomy, reducing the number of iatrogenic lesions 2

The experience with the use of ICG fluorescence in adults has shown multiple applications in recent years (colorectal, vascular, hepatobiliary, or tumor surgery) 3 ; however, the experience and bibliography described in pediatric cases are specific.

We present our experience in different laparoscopic procedures performed in children using ICG fluorescence imaging.

## Case Reports

A high-definition camera (10 mm) (Stryker) equipped with a visible infrared light source (800 nm) was used.

The laparoscopy camera used includes 3 CMOS chip technology, 1920 × 1080 p resolution, DVI, and S-VHS outputs and interval of 1/60 (1/50)-1/50000 seconds.

The ENV (endoscopic near-infrared visualization) mode is used as a light source.

### Case 1

**Fig. 1:**
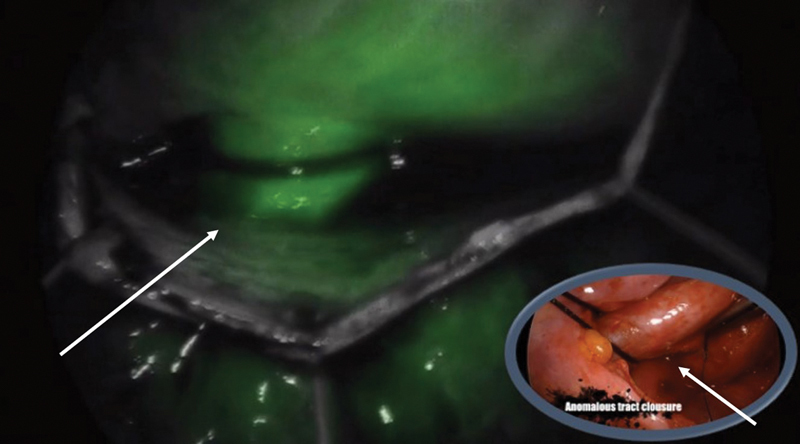
Aortocoronary fistula ligation. The image shows vascular permeability of the fistula, clearly demonstrated with the uptake of indocyanine green through it.

A 14-year-old girl presented with aortocoronary fistula, which caused a decreased coronary flow during diastole. Her clinical condition worsened during exercise. Right three-port (3 mm) thoracoscopy was performed in upright positions. The fistulous tract was readily identified and dissected on arrival at the right atrium. The presence of this rare vascular anomaly was confirmed by fluorescence by immediate injection of ICG (dose of 0.2 mg/kg), allowing better visualization and secure ligature ( Fig. 1 ).

### Case 2

A 13-year-old boy was scheduled for varicocelectomy. He had a clinical history of asymmetry and testicular pain. Umbilical, and right and left flank trocars (5 mm) were introduced for lens and instruments, respectively.

**Fig. 2:**
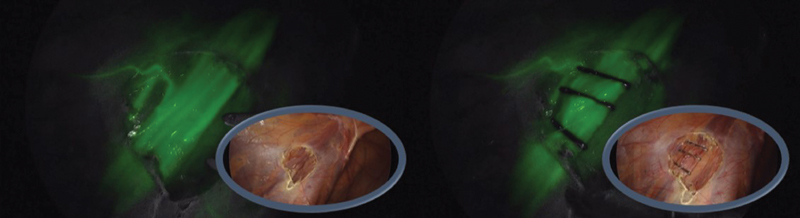
Ligation of spermatic vessels in varicocele. After the injection of the contrast, the vessels are filled (arterial and venous) and its correct ligature is verified, thanks to the infrared light of the fluorescence that indicates the vascular tree.

After intravenous (IV) injection of ICG, the arterial vessels were initially visualized following by the venous vessels. Thereafter, ligation of the spermatic cord was performed in block, ensuring selection of all vessels and avoiding the section of lymphatics that are not filled in this phase ( Fig. 2 ).

### Case 3

**Fig. 3:**
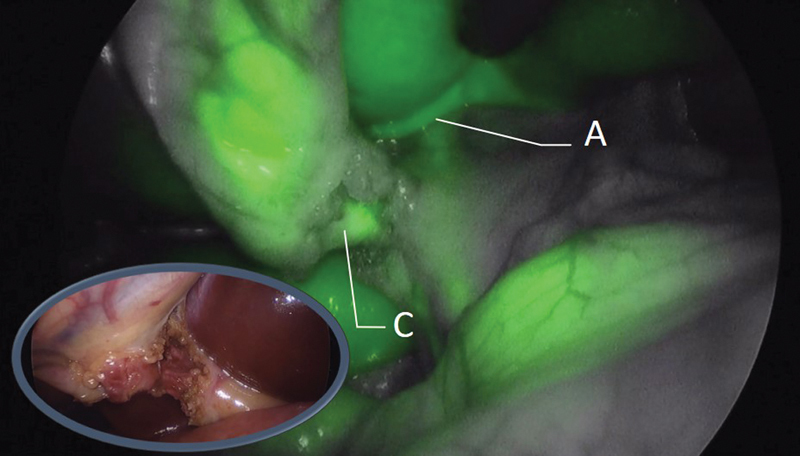
Cholecystectomy. Thanks to the fluorescence, contrast uptake can be observed initially in the cystic artery (A) and later in the cystic duct (C). In the image, we are in a late phase of fluorescence since both structures can be visualized.

A 13-year-old girl was admitted because of cholelithiasis and recurrent abdominal pain. She required two previous hospital admissions. Laparoscopic cholecystectomy was scheduled few days after admission. Fifteen minutes after ICG IV injection, the biliary tree was perfectly drawn, allowing clear identification of cystic artery, common bile duct, and hepatic duct. Safe dissection of the bile duct and artery was performed, completing cholecystectomy with total control of all surgical maneuvers ( Fig. 3 ).

### Cases 4 and 5

**Fig. 4:**
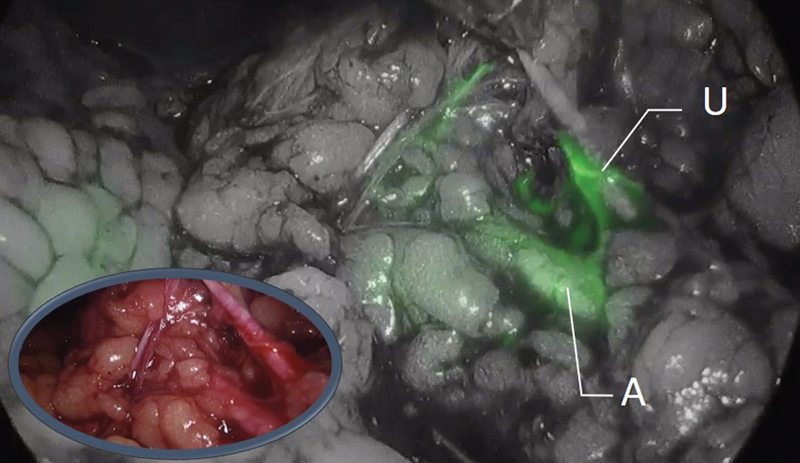
Nephrectomy. The image shows the renal artery (A) and periureteral vessels (U). Without fluorescence, the differentiation between the ureter and the vessels is difficult. Thanks to the fluorescence, we can identify them more easily since the ureter does not present contrast uptake.

Two children aged 3 and 6 years, respectively, had steroid-resistant hypertension and renal failure. Nephrectomy was indicated in each of them, which was performed by retroperitoneal laparoscopy. In both cases, intraoperative injection of indocyanine dye allowed renal vascular anatomy to be identified with certainty, showing the peripheral vascularization of the ureter. This technique definitively facilitated safe dissection of the renal hilum ( Fig. 4 ).

In all cases, we initially administrated ICG dye through a peripheral venous access at a standard dose of 0.2 mg/kg.

No adverse effects were present during or after IV ICG injections.

All patients were observed for 30 minutes to 1 hour in the recovery room, except the patient with aortocoronary fistula who was in the pediatric intensive care unit overnight.

## Discussion

ICG is an anionic molecule that is soluble in water, with a molecular mass of 776 daltons. After IV injection, ICG binds rapidly to plasma proteins, especially to lipoproteins (albumin).

Under near-infrared light, the released fluorescence can be detected using a specifically designed camera. 1

Not every laparoscopic equipment includes or is compatible for usage of an infrared light source, nor all equipment have the same technology for doing that.

We advise the use of devices that allow a vision with gray-scale functionality compared with those that only have black-and-white vision.

ICG has an exclusively biliary excretion; therefore, its most logical application in the visualization of biliary tree anatomy during laparoscopic cholecystectomy, 2 as shown in our case. After injection of ICG, the cystic artery could be initially observed, and 15 minutes later, the common hepatic, common bile duct, and cystic duct were identified. In addition, this allowed better visualization and anatomical dissection, avoiding injury to the biliary tree. This technique also avoids performing intraoperative cholangiography when bile duct injury is suspected during the procedure. 4

ICG has many other applications already described in the literature; it allows identification of sentinel node in breast tumors, melanoma, and prostate cancer among others. It also facilitates lymphadenectomy in tumors with lymphatic spread by local injection. 5

In colorectal surgery, it facilitates intestinal resections and is used to verify the adequate vascularization of the intestinal anastomoses, demonstrating a lower rate of postoperative complications. 6 7

Other applications have been described in surgery, such as liver resections, nephrectomies, and splenectomies. 8 9 In summary, ICG images are recommended for interventions in which visualization of the vascular anatomy is necessary to differentiate between anatomical and vascular variants, 3 10 as describe in our series of patients.

The ICG imaging system seems to be simple and safe. Its application in adult surgery is wide and contrasted. The ability to visualize the vascular structures or the bile duct anatomy allows us to approach laparoscopic techniques of different complexities with greater safety for the patient. We have verified its use in children. Larger and more specific studies are needed to confirm its applicability, expand its indications, and address its advantages and disadvantages.

## Notes

New Insights and the Importance for the Pediatric Surgeon The ability to visualize the vascular structures or the bile duct allows us to approach laparoscopic techniques of different complexities, with greater safety for the patient. We have verified its use in children.
